# Label-free multimodal nonlinear optical microscopy reveals features of bone composition in pathophysiological conditions

**DOI:** 10.3389/fbioe.2022.1042680

**Published:** 2022-11-22

**Authors:** Benedetta Talone, Arianna Bresci, Francesco Manetti, Federico Vernuccio, Alejandro De la Cadena, Chiara Ceconello, Maria Lucia Schiavone, Stefano Mantero, Ciro Menale, Renzo Vanna, Giulio Cerullo, Cristina Sobacchi, Dario Polli

**Affiliations:** ^1^ Department of Physics, Politecnico di Milano, Milan, Italy; ^2^ IRCCS Humanitas Research Hospital, Milano, Italy; ^3^ CNR-Institute for Genetic and Biomedical Research (CNR-IRGB), Milan, Italy; ^4^ Department of Clinical Medicine and Surgery, University of Naples Federico II, Naples, Italy; ^5^ CNR-Institute for Photonics and Nanotechnologies (CNR-IFN), Milan, Italy

**Keywords:** multimodal imaging, nonlinear microscopy, stimulated Raman scattering, bone and marrow composition, collagen, lipids

## Abstract

Bone tissue features a complex microarchitecture and biomolecular composition, which determine biomechanical properties. In addition to state-of-the-art technologies, innovative optical approaches allowing the characterization of the bone in native, label-free conditions can provide new, multi-level insight into this inherently challenging tissue. Here, we exploited multimodal nonlinear optical (NLO) microscopy, including co-registered stimulated Raman scattering, two-photon excited fluorescence, and second-harmonic generation, to image entire vertebrae of murine spine sections. The quantitative nature of these nonlinear interactions allowed us to extract accurate biochemical, morphological, and topological information on the bone tissue and to highlight differences between normal and pathologic samples. Indeed, in a murine model showing bone loss, we observed increased collagen and lipid content as compared to the wild type, along with a decreased craniocaudal alignment of bone collagen fibres. We propose that NLO microscopy can be implemented in standard histopathological analysis of bone in preclinical studies, with the ambitious future perspective to introduce this technique in the clinical practice for the analysis of larger tissue sections.

## 1 Introduction

Bone tissue is a specialized connective tissue made of a mineralized matrix in which the organic phase consists of collagen and a small amount of non-collagenous proteins hierarchically arranged, whereas the inorganic phase is mainly constituted by hydroxyapatite crystals. Overall, the amount and the spatial distribution of the diverse tissue components determine its mechanical properties (Ozasa et al., 2019), and alterations of bone microarchitecture and composition result in a variety of common and rare diseases, including osteoporosis and its opposite phenotype, i.e., osteopetrosis (Garnero, 2015).

Due to its peculiar structure and complexity, the study of the bone tissue is challenging. Among state-of-the-art techniques (Foessl et al., 2021), none can give insights into bone biochemical composition in a label-free way, preserving tissue integrity and spatial distribution of the diverse features.

A technology potentially overcoming current limitations is nonlinear optical (NLO) microscopy (Parodi et al., 2020a; Parodi et al., 2020b), which offers fast, high-specificity, and high-resolution imaging. NLO techniques combine morphological and functional/chemical information in a label-free fashion, enriching the single observation with multiple complementary contrast mechanisms and overcoming the fundamental limitations often imposed by fluorescent probes, such as cytotoxicity, poor binding specificity, and conflict with natural cellular functions (Jensen, 2012; Marchetti et al., 2019).

NLO microscopy exploits ultrashort (sub-picosecond duration) pulsed lasers in the near-infrared (NIR) wavelength region, thus allowing high penetration depth in tissues. Due to the nonlinear optical generation mechanism, these signals are generated almost exclusively at the focal point of the objective lens, thus overcoming the need for confocal pinholes to remove out-of-focus photons and providing intrinsic three-dimensional sectioning capability (Denk, 1996). Multimodal NLO imaging may exploit a variety of different contrast mechanisms such as two-photon excitation fluorescence (Oheim et al., 2006) (TPEF), second-harmonic generation (Chen et al., 2012) (SHG), and coherent Raman spectroscopy (CRS) (Polli et al., 2018).

**FIGURE 1:**
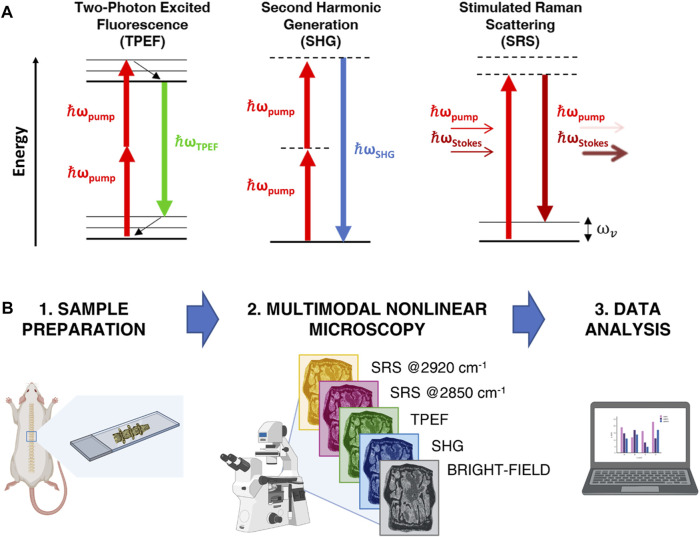
**(A)** Jablonski diagrams of TPEF, SHG and SRS processes. **(B)** Sketch of the experimental workflow.

TPEF is the archetypical multi-photon imaging technique. It is based on the physical mechanism whereby, when two incident photons are simultaneously absorbed by an electron, the latter is promoted from the ground state to an excited state (Figure 1A). After an initial ultrafast internal relaxation within the excited state, the electron radiatively returns to the ground state, thus emitting a photon with energy smaller than twice that of the excitation photons (Figure 1A). Compared to single-photon confocal imaging, TPEF microscopy reduces overall photobleaching and photodamage by confining it to the narrow region around the focal plane (Galli et al., 2014). Endogenous TPEF of intrinsic fluorophores, such as elastin, reduced Nicotinamide Adenine Dinucleotide (NADH) and Flavin Adenine Dinucleotide (FAD), yields physiological and pathological information from biological tissues at subcellular resolution in a completely label-free manner (Liu et al., 2018; Knyaz’kova et al., 2022).

SHG is a second-order non-linear process occurring when two photons at a certain frequency impinge on a non-centrosymmetric material of dimension comparable to the excitation wavelength, such as bundles of collagen fibers, and generate a new photon with twice the frequency and energy (Figure 1A) (Campagnola and Dong, 2011). Because of its polarization-dependent nature, SHG allows obtaining information about spatial distribution and orientation of the analyzed material (Stoller et al., 2002). While TPEF implies energy absorption in the specimen and radiates from the focal volume in any direction, SHG is a coherent process that does not involve any absorption and propagates collinearly with the excitation beam. TPEF and SHG techniques can be easily combined, being generated with the same laser source, and can be detected separately by applying proper spectral filtering.

Besides multi-photon microscopy techniques, also vibrational microscopy is having a great impact in the biological imaging field (Vanna et al., 2022a). This technique relies on the Raman scattering process and provides a label-free method of accessing the vibrational spectrum of materials and molecules (Vanna et al., 2022b). In this way, the chemical bonds of the molecules can be studied and identified with high biochemical specificity. Stimulated Raman scattering (SRS) along with coherent anti-Stokes Raman scattering (CARS) are the most widely used CRS techniques (Volkmer et al., 2009; Cheng et al., 2022; De la Cadena et al., 2022; Vernuccio et al., 2022). With respect to spontaneous Raman, CRS techniques guarantee high-speed detection thanks to the coherent excitation of the vibrational modes at the sample plane. Both SRS and CARS consist of exposing a sample to two temporally and spatially overlapped laser pulses of different frequencies, $\omega_{p}$ and $\omega_{s}$ , called pump and Stokes, respectively. When the difference $\omega_{p}$ - $\omega_{s}$ is equal to a vibrational mode Ω of the sample, the Raman signal is generated and amplified by the simultaneous in-phase vibration of all molecules in the focal volume (Nandakumar et al., 2009). In SRS, the coherent interaction of pump and Stokes beams with the specimen induces the excitation to a virtual state with consequent stimulated emission to the vibrational level of interest. In this process, a Stokes photon is generated, i.e., the Stokes experiences a stimulated Raman gain (SRG), and simultaneously a pump photon is annihilated, i.e., the pump undergoes a stimulated Raman loss (SRL), as shown in Figure 1A. Moreover, since the generated SRS signal is directly proportional to the concentration of scatterers in the focal volume, it is a valuable solution for extrapolating quantitative chemical information out of the sample also at very poor concentration of analytes, which is usually the case for biological matter. In SRS modality, the SRG is detected as a small variation on top of the orders of magnitude larger Stokes beam intensity. This, paired with the use of compact, cost-effective but noisy fiber laser, presents challenges for real microscopy applications, which can be overcome with balanced detection schemes, providing almost shot-noise limited performances (Kumar et al., 2012; Riek et al., 2016; Crisafi et al., 2017; Cheng et al., 2021).

Owing to its properties, multimodal NLO imaging has been recently applied to diverse biological contexts (Parodi et al., 2020b), and, in the musculoskeletal tissue, to the development of articular cartilage (He et al., 2017) and skeletal muscle (Sun et al., 2014). Single-modality TPEF imaging was exploited to obtain histological information of bone architecture, along with indication of osteoarthritis, osteomyelitis, and malignancy condition from unstained bone (Yoshitake et al., 2022). Similarly, single-channel third-harmonic generation (THG) microscopy enabled label-free imaging of bone porosity and interfaces (Genthial et al., 2017). On the other hand, simultaneous SHG, THG, CARS, and TPEF nonlinear optical microscopy was tested to image small areas (i.e., 250 × 250 µm) of a canine bone femur, deriving qualitative information about phosphate mineralization, collagen, and bulk morphology (Kumar et al., 2015). Thanks to the short pixel dwell time of 5 ms, fast multimodal microscopy was proved effective for perspective rapid and quantitative investigations concerning relevant biomedical questions in bone research.

We exploited the potential of multimodal NLO microscopy to analyze characteristics of bone composition in a quantitative approach, imaging entire vertebrae of murine models. We hypothesized that, owing to its non-disruptive nature, multimodal NLO microscopy images on large tissue areas might provide new hints into tissue composition and spatial organization, which are inherently linked to major mechanical properties.

In the present work, we applied multimodal NLO microscopy to murine spine sections from either a wild-type (WT) mouse or a genetic mouse model showing bone loss, i.e., the Dpp3 Knock-out (Dpp3 KO) mouse (Menale et al., 2019). The dipeptidyl peptidase 3 (Dpp3) is a ubiquitous zinc-dependent aminopeptidase involved also in the Keap1/Nrf2 antioxidant signaling pathway. Mice lacking DPP3 (Dpp3 KO) present sustained oxidative stress and inflammation in the bone microenvironment, overall resulting in bone loss. In humans, recent findings in post-menopausal osteoporotic women supported the critical role played by DPP3 in bone homeostasis and tissue health.

Figure 1B provides a schematic representation of the analytical workflow. Briefly, spine sections were obtained from WT and Dpp3 KO mice counterparts, then examined under a multimodal custom-built microscope, (Crisafi et al., 2018), operating in four different modalities: bright field, SHG, TPEF and SRS. This latter was applied to image our samples at both 2850 cm^−1^ and 2920 cm^−1^ Raman shifts, corresponding to the main vibrational frequencies of lipids and proteins, respectively. Data were analyzed in the Fiji-ImageJ software (Rueden et al., 2017) and in Python using Numpy (Harris et al., 2020), Scipy (Virtanen et al., 2020) and the “NanoImagingPack” library: https://gitlab.com/bionanoimaging/nanoimagingpack This led us to uncover different biochemical and structural traits in a single image, to quantify them, and to highlight significant differences between bone loss models and control counterparts. Our findings demonstrate that multimodal NLO microscopy performed on large tissue areas is an effective tool for the fast characterization of bone composition, without the need for time-consuming, destructive, or perturbative sample preparation.

## 2 Materials and methods

### 2.1 Animals

Mice in which the Dpp3 gene was ubiquitously inactivated (Dpp3 KO) have been previously described (Menale et al., 2019). Dpp3 KO and WT mice were group-housed in a specific-pathogen-free animal facility, under a 12-h dark/light cycle, with water and food provided *ad libitum*.

All the procedures involving mice were performed in accordance with the ethical rules of the Institutional Animal Care and Use Committee of Humanitas Research Hospital and with international laws (Italian Ministry of Health, protocol n.07/2014-PR).

### 2.2 Tissue samples preparation

Mice were euthanized by CO_2_ asphyxiation; tissues were harvested and fixed in 4% paraformaldehyde (PFA). Bones were processed for embedding in methyl methacrylate (MMA) immediately after fixation, without any decalcification. Sections of MMA-embedded lumbar spine of three different mice per genotype were laid on polylysine-coated fused silica slides, unplasticized and analyzed by multimodal NLO microscopy. On each section, the entire area of a vertebra was analysed.

### 2.3 Multimodal nonlinear optical microscopy

**FIGURE 2:**
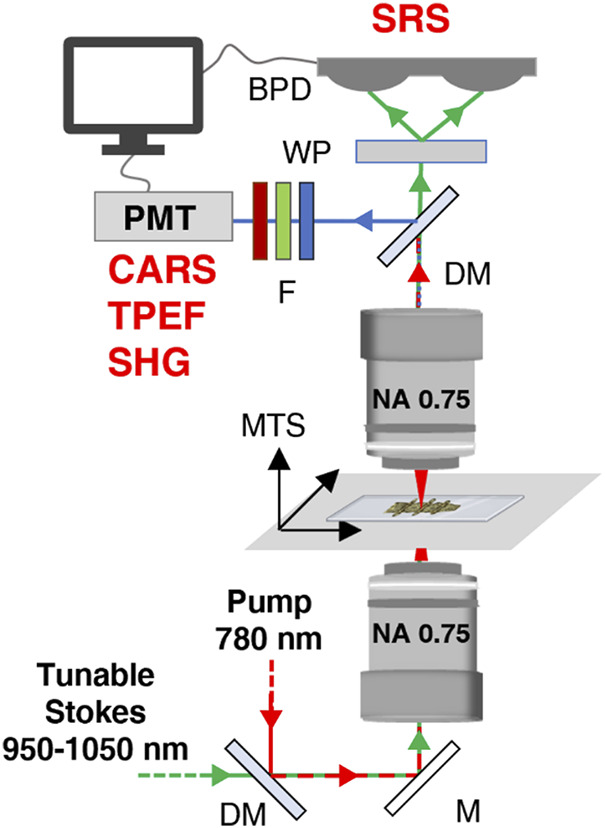
Schematic representation of the microscope setup. DM, dichroic mirror; M, mirror; MTS, motorized translation stage; F, filter; WP, Wollaston prism; PMT, photomultiplier tube; BPD, balanced photodiode; NA, numerical aperture.

We developed a multimodal nonlinear optical microscope, assembled with off-the-shelf components, able to perform imaging in four different modalities: linear transmission, two-photon excitation fluorescence (TPEF), second harmonic generation (SHG), and stimulated Raman scattering (SRS). We employed a multi-branch Erbium-doped amplified fiber laser to generate a pump beam at 780 nm and a tunable Stokes beam, in the 930–1060 nm range with a repetition rate of 40 MHz. In this way, the CH-stretching region (2800–3100 cm^−1^) of the Raman spectrum is fully covered for SRS imaging. The sole pump beam is used for TPEF and SHG microscopy. Our home-built microscope has an inverted transmission configuration. For excitation and detection, we used two identical Zeiss objectives (×100 magnification, 0.75 numerical aperture, 4-mm working distance, field number 25). The samples were mounted on a three-axis motorized translation stage, made up by a vertical (Z) stage (Mad City Labs Inc: model MMP1) to adjust the focus and a two-axis stage (Standa: model 8MTF-102LS0) along the (X-Y) sample plane to perform raster scanning of the image. A dichroic mirror was used to split the generated nonlinear signals. The TPEF/SHG photons were sent to a photomultiplier tube (Hamamatsu: model R3896) and extracted applying the appropriate set of filters: for the TPEF modality, a FESH0600 (Thorlabs) short-pass filter is used, since it possesses a good transmittance in the 400–600 nm range, thus matching the spectral range of both NADH and FAD autofluorescence (Ruofan et al., 2020), while also rejecting the unwanted Second-Harmonic Generation signal from collagen; for the SHG modality, the FF01-390/18–25 (Semrock) bandpass filter allows for selecting the narrow SHG signal from collagen, centred at 390 nm. In addition, for both modalities, the following combination of filters are also employed to block the fundamental beams: NF785/33, FESH0700 and FESH0750 (Thorlabs) The SRS signals were detected using the in-line balanced detection scheme (Crisafi et al., 2017), so that the transmitted SRS and Stokes photons are sent to a balanced photodiode, which features a responsivity above 0.4 A/W at our wavelengths of interest (Thorlabs: model PDB210A/M). A schematic representation of the microscope is reported in Figure 2. For additional information, the reader can refer to the work of Crisafi and colleagues (Crisafi et al., 2018).

### 2.4 Image acquisition setting

One complete vertebra from each sample was imaged in two consecutive acquisitions comprising four different modalities: linear light transmission, TPEF, SHG, and SRS at 2850 and at 2920 cm^−1^ Raman shifts, resonant with lipids and proteins, respectively (Yoshitake et al., 2022). To account for polarization-dependent signal generation in the SHG modality, we compared the results obtained with the pump beam polarization oriented either parallel or perpendicular to the craniocaudal axis of the spine. The laser power was kept constant at 20 mW for the pump beam and 1.2 mW for the Stokes beam. Images were acquired with a pixel dimension of 1 × 1 µm and a pixel dwell time of 5 ms.

### 2.5 Analysis of collagen content

In each SHG image of WT and KO samples, we defined a region of interest (ROI) comprising only either the cortical or the trabecular bone, disregarding the bone marrow, intervertebral disc, growth plate and areas outside the vertebra (see Supplementary Figure S1). We then computed the average raw SHG pixel intensity over these two ROIs using the Fiji-ImageJ software (Rueden et al., 2017). As SHG signals scale linearly with the density of the scatterers in the voxel, we obtained data that represent the average density of collagen in the cortical and trabecular bone, not affected by their relative extension.

### 2.6 Analysis of collagen fibres orientation—Sobel algorithm

We applied a Sobel filter implemented in Python to the two ROIs of the cortical and trabecular bone described in the previous paragraph. This algorithm consists in cross correlating the SHG images with the following two kernels:

$$\begin{array}{c} G_{x}=\left[\begin{array}{ccc} +1 & 0 & -1\\ +2 & 0 & -2\\ +1 & 0 & -1 \end{array}\right],\\ G_{y}=\left[\begin{array}{ccc} +1 & +2 & +1\\ 0 & 0 & 0\\ -1 & -2 & -1 \end{array}\right]. \end{array}$$ (1)

This allowed us to compute the pixel-wise angular orientation Θ of the collagen fiber pattern as (Nixon and Aguado, 2020):

$$\Theta =\arctan\left(\frac{G_{y}}{G_{x}}\right).$$ (2)

### 2.7 Primary calvaria osteoblast culture

The upper part of the skull (denominated calvarium) was dissected from WT (*n* = 4) and Dpp3 KO (*n* = 6) newborn mice (postnatal day 3–4), cleaned from adherent soft tissue, and then sequentially digested in 1x HBSS (EuroClone SpA) containing 1 mg/ml collagenase type IV (Sigma-Aldrich), 0.025% Trypsin (EuroClone SpA) and 1% Penicillin/Streptomycin (P/S), at 37°C. The cells from digests 2–4 were collected, pooled, and washed, then plated in α-MEM (Sigma-Aldrich) supplemented with 15% Fetal Bovine Serum (FBS, Lonza) and 1% P/S.

### 2.8 Analysis of *in vitro* collagen production

Primary osteoblasts (50,000 cells/cm^2^ in 12-well plate) were treated with 50 μg/ml of ascorbic acid for 8 h. Then, the cells were fixed with 4% PFA for 20 min at room temperature (RT), stained with 0.1% Sirius red (Sigma Aldrich) in saturated picric acid for 4 h and washed with PBS (Lonza). The stain was solubilized in 300 μL of destain solution (0.2 M NaOH/methanol 1:1) and the optical density was measured at 540 nm with a Synergy™ H4 instrument (BioTek Instruments, Inc.).

### 2.9 Gene expression analysis

Total RNA was extracted from primary osteoblast cell cultures using the PureZOL™ Reagent (Bio-Rad), following the manufacturer’s instructions. For RNA extraction from murine flushed long bones (*n* = 4 WT, 7 KO), the frozen tissue was crashed in a mortar, then transferred in tubes and homogenized in a TissueLyser II instrument (Qiagen) in the presence of PureZOL™ Reagent; afterwards, standard procedures were applied. Reverse transcription was carried out using 1.0 μg total RNA and High-Capacity cDNA Reverse Transcription Kit (Applied Biosystems). Quantitative PCR (qPCR) was performed using SsoAdvanced™ SYBR^®^ Green Supermix (Bio-Rad) and gene-specific primers as detailed in Table 1. The amplification was performed using the ViiA7 Real-Time PCR Detection System (Applied Biosystems) with the following cycling conditions: cDNA denaturation and polymerase activation step at 95°C for 20 s followed by 40 cycles of denaturation at 95°C for 1 s and annealing at 60°C for 20 s; extension step for 60 cycles at 65°C for 30 s and melting curve analysis step at 65°C–95°C with 0.5°C increment for 2 s/step. The relative gene expression analysis of target genes was conducted following the comparative 2^−ΔΔCT^ method and the normalized expression was calculated as arbitrary units (AU) in comparison with pertinent controls.

**TABLE 1:** Primers used for qPCR analysis of RNA extracted from primary osteoblast cell cultures.

Gene name	Gene symbol	Primers
CD36 Molecule	CD36	F: 5′-TTG​AAA​AGT​CTC​GGA​CAT​TGA​G 3′
		R: 5′-TCA​GAT​CCG​AAC​ACA​GCG​TA-3′
LDL Receptor Related Protein 1	Lrp1	F: 5′-CTG​AGC​CTG​CCG​ACA​ATG​A-3′
		R: 5′-TTG​GTA​CAG​TTG​GCG​TGT​GT-3′
Fatty Acid Transporter, Member 1	Fatp1	F: 5′-ATC​TGA​TGC​GGG​CTC​CTG 3′
		R: 5′-AAC​AGA​GAG​GCC​AAA​GAG​GT-3′
Fatty Acid Binding Protein 4	Fabp4	F: 5′-GAC​AAG​CTG​GTG​GTG​GAA​TGT-3′
		R: 5′-ATC​CAG​GCC​TCT​TCC​TTT​GG-3′
Peroxisome Proliferator-Activated Receptor Gamma Coactivator 1-Alpha	Pgc1	F: 5′- CTT​GCT​AAC​ATC​AGA​GAG​GAT​ATC​TTG-3′
		R: 5′- GGCAGTTCAACCCCGA-3′
Carnitine Palmitoyltransferase 1A	Cpt1A	F: 5′- GACTCCGCTCGCTCATTC -3′
		R: 5′-TCT​GCC​ATC​TTG​AGT​GGT​GA -3′
Peroxisomal Biogenesis Factor 7	Pex7	F: 5′-GCA​CAC​GCA​GGA​GGT​GTA​TAG-3′
		R: 5′-GAT​CCC​ATG​AGC​CAG​ACA​CAA-3′
Glucose Transporter Type 1	Glut1	F: 5′-AGC​CCT​GCT​ACA​GTG​TAT-3′
		R: 5′-AGG​TCT​CGG​GTC​ACA​TC-3′
Sirtuin 1	Sirt1	F: 5′-TGA​TTG​GCA​CCG​ATC​CTC​G
		R 5′-CCA​CAG​CGT​CAT​ATC​ATC​CAG
Collagen type I alpha 1 chain	Col1a1	F: 5′-CTC​CTG​GTA​TTG​CTG​GTG​CT-3′
		R: 5′-TTC​ACC​AGG​AGA​ACC​TTT​GG-3′
Glyceraldehyde-3-phosphate dehydrogenase	Gapdh	F: 5′-AGG​TCG​GTG​AAC​GGA​TTT​G-3′
		R: 5′-TGT​AGA​CCA​TGT​AGT​TGA​GGT​CA-3′

### 2.10 Statistics

Statistical analysis for all the experimental data obtained was performed using the non-parametric Mann–Whitney *U* test (GraphPad Prism 5.0; GraphPad Software, Inc.). Statistical significance was considered where *p* < 0.1 (90% CI). All data are presented as mean ± standard error of the mean (SEM).

## 3 Results and discussion

### 3.1 Multimodal nonlinear imaging

**FIGURE 3:**
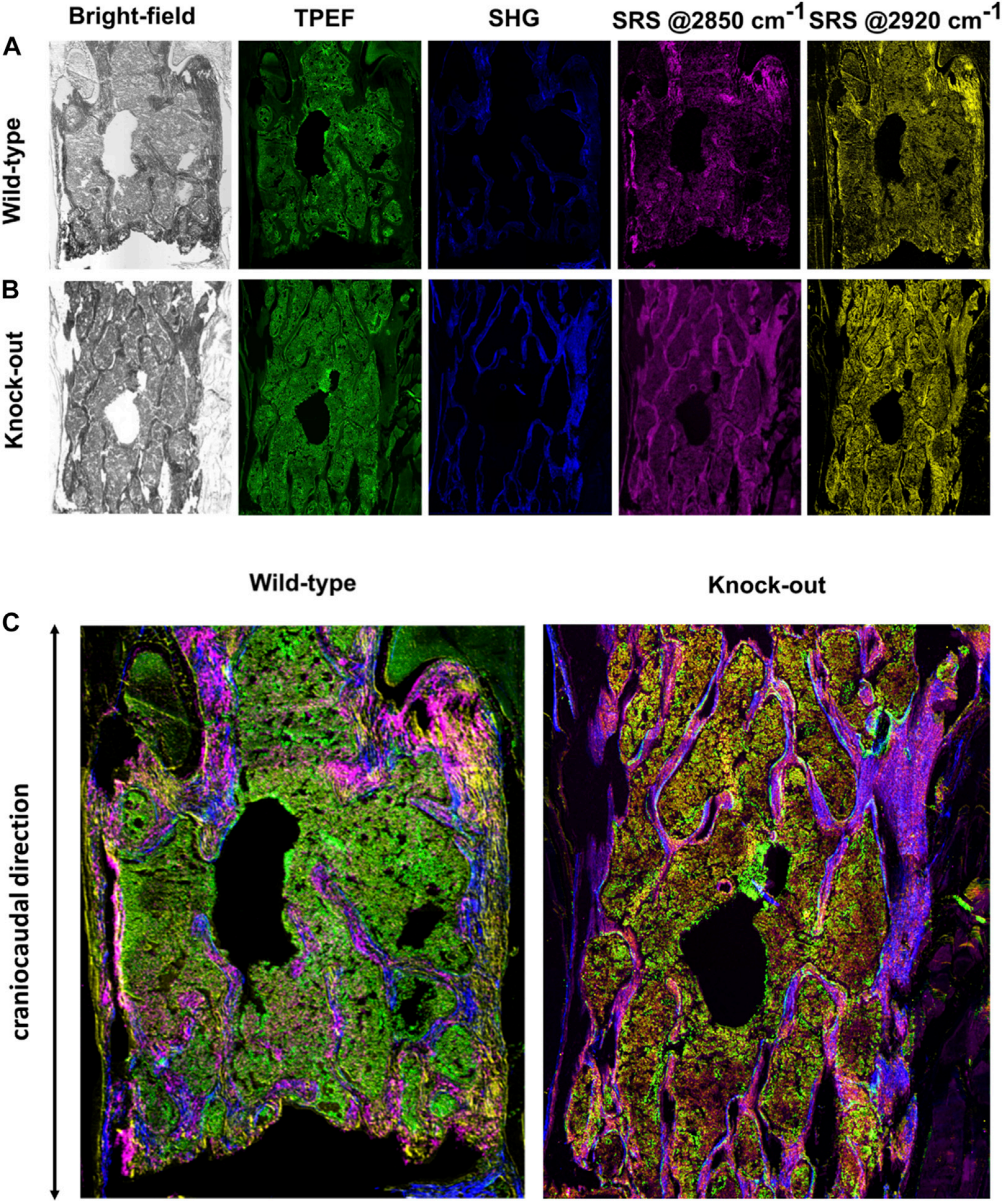
**(A)** Complete set of images for a representative WT sample. **(B)** Complete set of images for a representative KO sample. **(C)** Multimodal images as result of combination of images reported in panels **(A)** and **(B)**.

The new multimodal NLO microscope that we developed as described above, was able to map the major biochemical components that constitute the murine bone over a large field of view (in the scale of few $mm^{2}$ ) spanning the whole section of a murine vertebra and in a label-free manner. Figures 3A,B show two complete sets of images acquired from a WT and a KO murine bone sample, respectively, using the four complementary imaging modalities, each one providing distinct information about the sample.

The bright-field images, obtained by recording the linearly transmitted Stokes beam, allow us to derive morphological information about the density of biological matter under observation. In the TPEF modality (green channel in Figure 3), the signal is generated from the excitation of intrinsic intracellular fluorophores such as NADH and FAD. Their presence is an indicator of oxidative and glycolytic metabolic mechanisms occurring at the cellular level (Zipfel et al., 2003). The SHG signal (blue in Figure 3) derives from the non-centrosymmetric structure and the remarkable second-order nonlinear susceptibility of collagen fibers (Kröger et al., 2021). In SRS modality, we could image the concentration of lipids and proteins (magenta and yellow in Figure 3, respectively) detecting their high-energy vibrational response at 2850 and 2920 cm^−1^, resonant with the CH_2_ and CH_3_ stretching modes, respectively (Ji et al., 2013).

We then combined the four NLO channels into multicolor images (Figure 3C), to investigate the co-localization of the multiple biological species on the same distribution map, thus obtaining a richer and more thorough understanding about the overall tissue composition. For example, combining the SRS signal of proteins at 2920 cm^−1^ and the SHG signal of collagen fibers, it is possible to recognize trabecular and cortical bone tissue, due to their dense content of proteins and collagen in the extracellular matrix.

### 3.2 Analysis of collagen content

We employed SHG microscopy to study the collagenous architecture of bone in terms of collagen amount and directionality. The former can be directly measured considering that collagen content is proportional to the SHG signal generated (James and Campagnola, 2021). The latter can be estimated thanks to the fact that SHG radiation possesses a polarization-sensitive nature: defining θ as the angle between the polarization direction of the pump field and the molecular axis of a collagen fiber, the induced local dipole moment varies with $\cos^{2}\theta$ , whereas the total radiation power scales with $\cos^{4}\theta$ (Moreaux et al., 2000). As a consequence, the generated signal is higher when the polarization of the excitation beam is oriented along the longitudinal axis of collagen fibers, acting as radiating dipoles, with respect to the opposite case when light is polarized along their transversal direction (Mostaço-Guidolin et al., 2017). Therefore, we performed experiments with the pump beam polarization oriented either parallel or perpendicular to the craniocaudal axis of the murine spine (Figure 3C). We used linearly polarized rather than circularly polarized light for SHG measurements to simultaneously acquire, in our multimodal approach, the SRS signal exploiting an in-line balanced detection for noise reduction (Crisafi et al., 2017).

#### 3.2.1 Collagen amount

**FIGURE 4:**
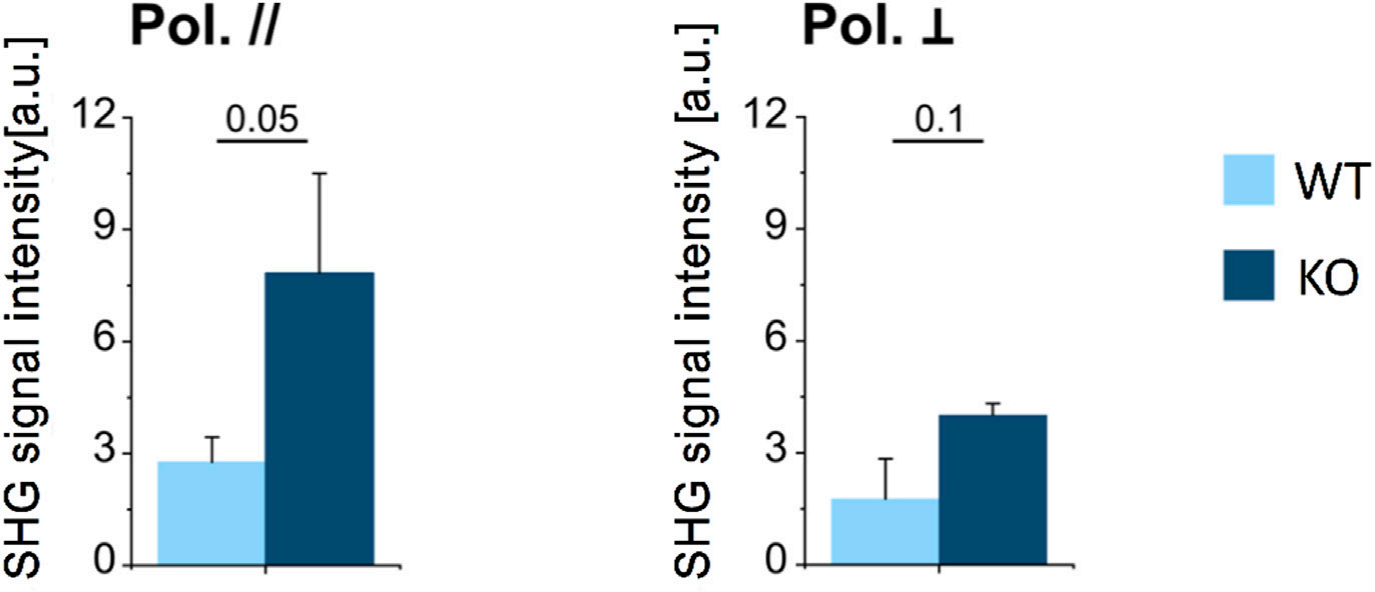
Average SHG signal intensity in mineralized bone (cortical and trabecular bone) in WT and KO mice, for parallelly- (left) and perpendicularly (right) polarized excitation laser field. Data were analysed using *t* test (*n* = 3 for both genotypes); *p* values are indicated above each bar plot.

We estimated the average density of collagen in the whole section of a vertebra, manually excluding the bone marrow from the mineralized osseous region (i.e., cortex and trabeculae, see Supplementary Figure S1) *via* the average SHG signal intensity, for the WT and KO models, measured under the same experimental conditions in terms of laser power, spot size and pulse duration. Results are reported in Figure 4, for parallel and perpendicular polarization of the excitation light with respect to the craniocaudal axis of the murine spine. For both polarizations, the amount of SHG signal was significantly higher in the KO model, suggesting that collagen content is greater in the KO compared to WT bone (see also Supplementary Figure S2).

**FIGURE 5:**
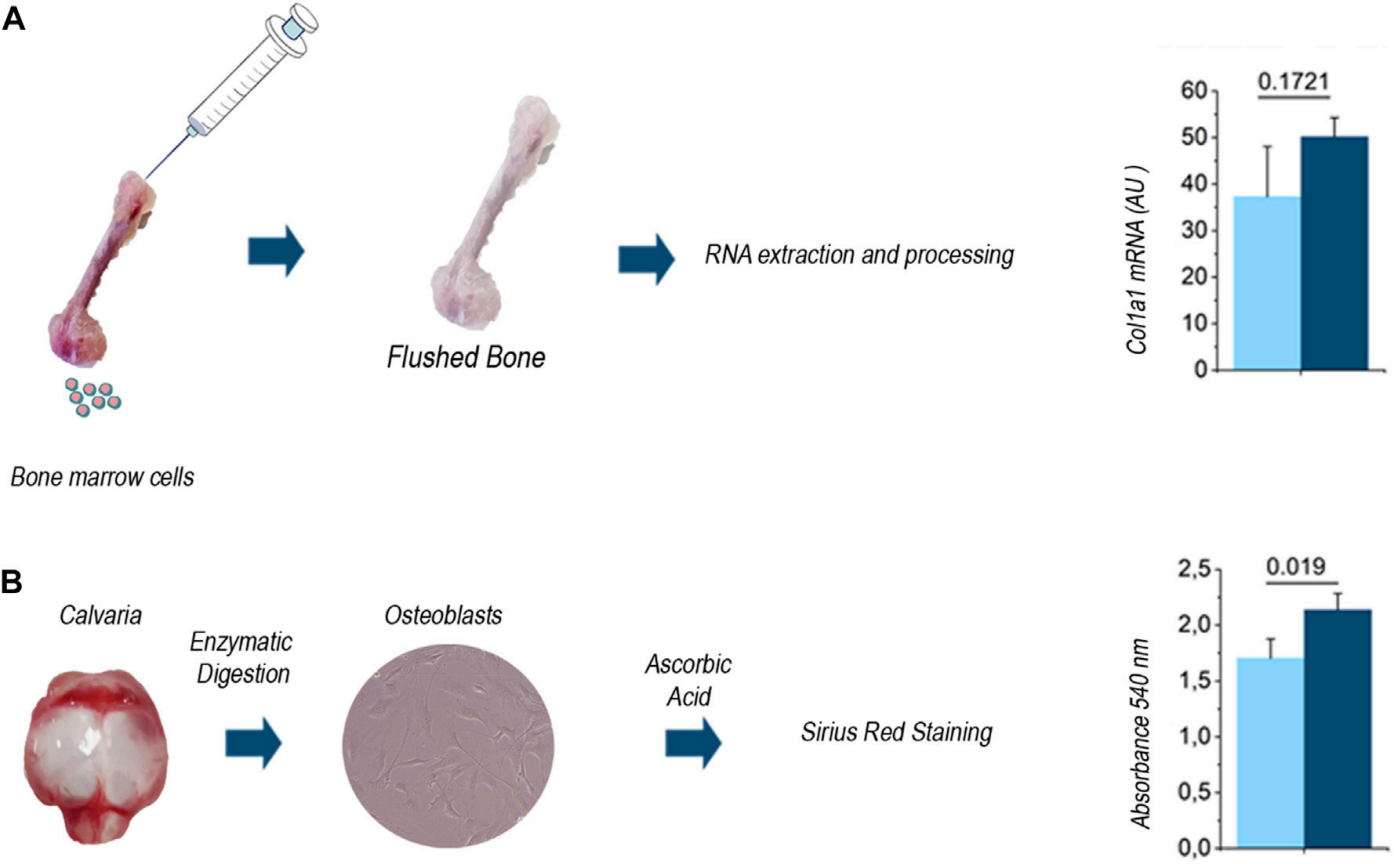
**(A)** Schematic representation of the tissue processing for RNA extraction and quantization of ColIa1 gene expression level in the WT and Dpp3 KO (*n* = 4 WT and *n* = 7 KO). **(B)** Schematic representation of the procedure for primary osteoblast isolation from WT and Dpp3 KO neonatal calvaria, and assessment of *in vitro* collagen production (*n* = 4 WT and *n* = 6 KO). Statistical analysis was performed using Mann Whitney test; *p* values are indicated above each bar graph.

Then we assessed whether standard investigational procedures in bone biology provided results in line with this evidence. Indeed, Menale et al. (2019) who generated and characterized the Dpp3 KO mouse model, found higher ColIa1 gene expression during *in vitro* osteoblast differentiation, as well as in cultures of primary osteoblasts from Dpp3 KO as compared to WT mice. As a further support, here we assessed ColIa1 gene expression in the total RNA extracted from Dpp3 KO and WT flushed bone and found an overexpression trend in the absence of Dpp3 (Figure 5A). We also evaluated *in vitro* collagen production from Dpp3 KO and WT primary osteoblast cultures, through Sirius-red staining and quantization, and found higher collagen production in KO than in WT osteoblast cultures from primary osteoblasts (Figure 5B). Overall, these results agree with data from SHG images and suggest that the lack of Dpp3 is associated with an increased collagen content in the bone tissue.

#### 3.2.2 Analysis of collagen directionality

**FIGURE 6:**
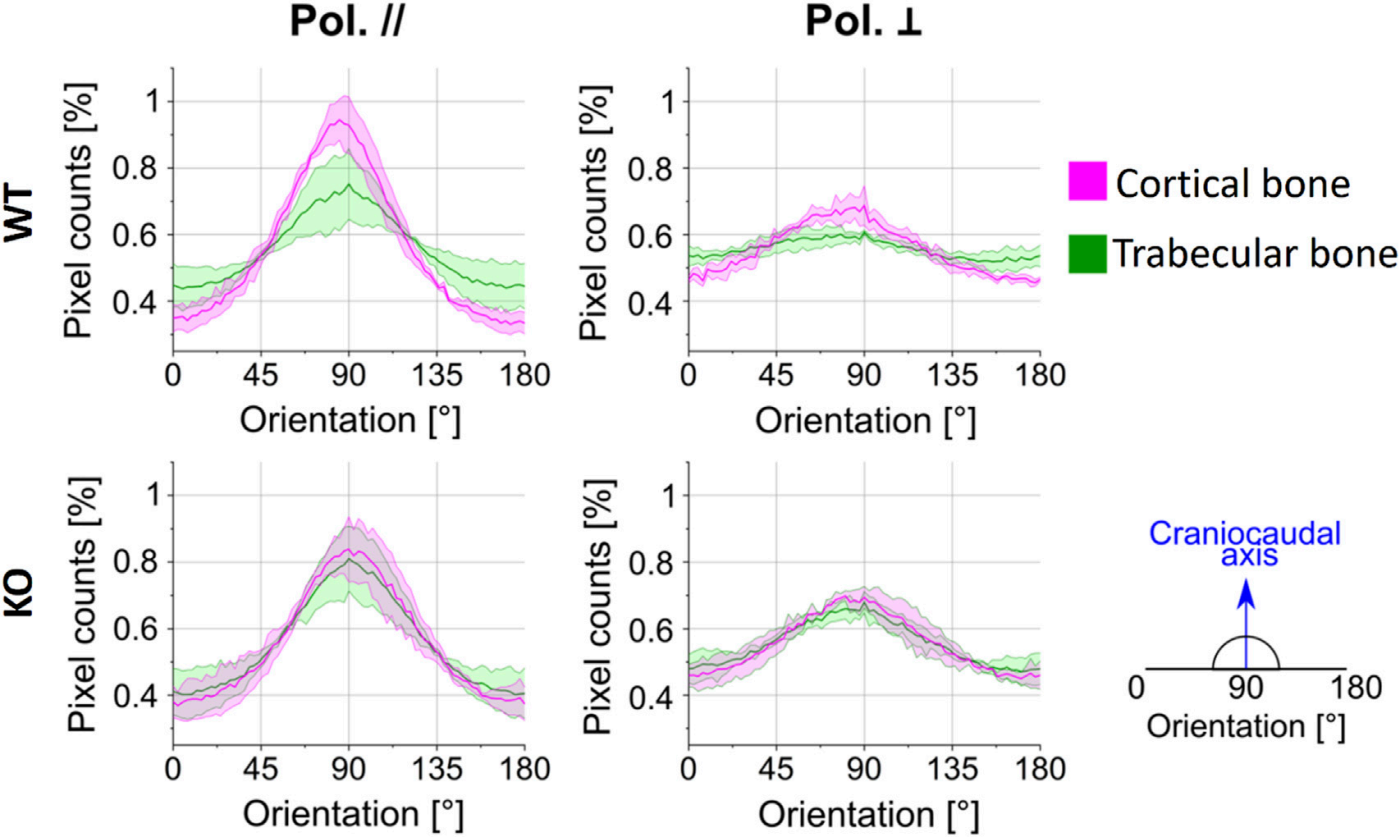
Collagen fiber orientation results: the normalized pixel counts are reported in percentage as a function of the fiber orientation in the 0°–180° range, for any combination of polarization’s orientation (parallel and perpendicular), sample model (WT and KO; *n* = 3 per genotype) and bone area (cortical and trabecular bone). The solid line represents the average value, and the shaded area the standard deviation.

We evaluated the directionality of collagen fibers thanks to the optical properties of polarization-sensitive SHG signals previously described. Figure 6 reports the pixel counts, in percentage, as a function of the fiber orientation in the 0°–180° range, employing the Sobel operator (see Methods), for the WT and Dpp3 KO mice. Results obtained for cortical and trabecular regions are plotted in magenta and green, respectively. As clear from the position of the Gaussian centers in terms of angular orientation [°], collagen fibers exhibit a prevalent orientation at 90°, i.e., along the craniocaudal axis of the murine spine, irrespective of the genotype and of the parallel/perpendicular polarization of the electric field of the excitation laser beam. This is, in line with the previous observation that the pixel counts are higher when parallel-polarized light is employed: the polarization of the impinging field matches the prevalent direction of fibers in the samples, thus maximizing SHG photon conversion.

Interestingly, the tissue sections of WT mice show a difference in collagen orientation between the cortical (magenta line) and the trabecular bone (green line) in both light polarizations (first row in Figure 6, as indicated by the fact that the curve of the pixel counts for the cortical and trabecular bone do not overlap). On the other hand, the tissue sections of Dpp3 KO mice (second row in Figure 6) exhibit a comparable fiber orientation in cortical and trabecular regions in both the conditions of light polarization, as indicated by the fact that the curve of the pixel counts for the cortical and trabecular bone are almost completely overlapping. Therefore, in the WT the normalized amount of collagen fibers aligned along the craniocaudal direction is higher in the cortical then in the trabecular region, while in the absence of Dpp3 there is no difference.

We modeled the data presented in Figure 6 as the sum of a baseline B, representing the portion of collagen fibers randomly oriented in any direction, and a Gaussian function with A representing the peak-baseline amplitude:

$$f=B+Ae^{-{\left(\frac{\theta -\theta_{0}}{\sqrt{2}\sigma }\right)}^{2},}$$ (3)

where θ_0_ is the preferential orientation of the fibers and σ its standard deviation. The values of these parameters and the corresponding coefficient of determination (*R* ^2^) of the fit are presented in Table 2 and Table 3, for polarization oriented parallel and perpendicular to the craniocaudal direction, respectively. As an indicator of the degree of orientation of the collagen fibers, we also reported in the Tables the percentage alignment ratio, defined as AR = A_G_/(A_G_ + A_B_), where A_G_ is the area beneath the Gaussian curve (considering an offset value equal to zero), which is associated with fibers aligned along the vertical direction, and A_B_ is the area underlying the baseline B. Randomly oriented fibers should display a value close to 0%, while this ratio should approach 100% for samples where all fibers are aligned in a single direction.

**TABLE 2:** Optimal parameter values that maximize the R2 of the fit for data collected with the pump beam parallel-polarized to the craniocaudal axis of the spine.

	WT		KO	
	Cortical bone	Trabecular bone	Cortical bone	Trabecular bone
A	0.60	0.30	0.46	0.39
θ0	85°	89°	91°	91°
σ	25.4°	27.6°	29.0°	28.3°
R (Garnero, 2015)	0.99	0.99	0.99	0.99
B	0.33	0.44	0.37	0.40
Alignment Ratio (AR, %)	24.4	11.6	20.0	16.1

**TABLE 3:** Optimal parameter values that maximize the R2 of the fit for data collected with the pump beam perpendicular-polarized to the craniocaudal axis of the spine.

	WT		KO	
	Cortical bone	Trabecular bone	Cortical bone	Trabecular bone
A	0.21	0.08	0.24	0.19
θ0	79°	73°	85°	83°
σ	34.6°	35.3°	31.1°	33.2°
R (Garnero, 2015)	0.99	0.95	0.99	0.99
B	0.46	0.52	0.45	0.47
Alignment Ratio (AR, %)	9.92	3.64	10.3	8.55

Comparing the parameters reported in Table 2 and Table 3, we can conclude that: 1) When exciting the sample with light polarized parallel to the craniocaudal axis, the baseline B is ≈23% smaller (0.39 instead of 0.48 on average) with respect to the opposite condition of illumination with light polarization perpendicular to this direction. This indicates that the collagen fibers are predominantly oriented in the vertical direction. 2) Similarly, the standard deviation σ of the Gaussian distribution is ≈ 22% smaller (27.58° instead of 33.59° on average) when the SHG is excited with light polarized parallel to the craniocaudal axis, suggesting a predominant collagen fiber distribution along this direction. Instead, using the pump beam with polarization oriented perpendicularly, the minor portion of collagen fibers oriented perpendicular to the craniocaudal axis will be more efficiently excited. Accordingly, the alignment ratios AR (Table 2 and Table 3) are higher in the case of the parallel-polarized pump. Furthermore, the AR in the cortical is more than 2-fold higher than the one in the trabecular bone, especially for WT samples, for both polarizations. This result quantitatively distinguishes the cortical regions from the trabecular ones, in terms of collagen fibers degree of orientation. This is not the case for KO samples, where the AR is only slightly higher in the cortical than in the trabecular bone, for both polarizations. This can be appreciated also in Figure 6, where the plots describing collagen orientation in KO models in the cortical and trabecular bone appear to be overlapped.

In conclusion, in WT sample, the cortical and the trabecular regions feature differences in terms of collagen fiber orientation, due to the physiological different specialization of these bone areas: the cortex, featuring the highest AR values, is the main responsible for vertebrae mechanical strength and load bearing. Conversely, in KO samples, cortical collagen fibers are not strongly oriented along the craniocaudal direction. We speculate that this altered spatial distribution might result in reduced mechanical properties of the KO bone as compared to WT bone.

### 3.3 Lipid and protein content in bone and bone marrow

In WT murine spines, the images generated in the SRS modality at 2850 cm^−1^ (magenta in Figure 3A) and 2920 cm^−1^ (yellow in Figure 3A) show that both the lipid and protein content of the tissue appeared slightly concentrated in the mineralized areas, i.e. cortical and trabecular bone, as compared to the marrow compartment. Conversely, in the KO samples the protein signal at 2920 cm^−1^ (yellow in Figure 3B) was present in the whole field of view and distributed evenly in the bone and bone marrow, with a small predominance in the former. Interestingly, the lipid SRS signal at 2850 cm^−1^ showed a more distinctive distribution, also compared to the signals detected in the WT sample. In fact, the lipid content appeared to be dominant in bone, in both the cortical and the trabecular part, differentiating it from the bone marrow.

**FIGURE 7:**
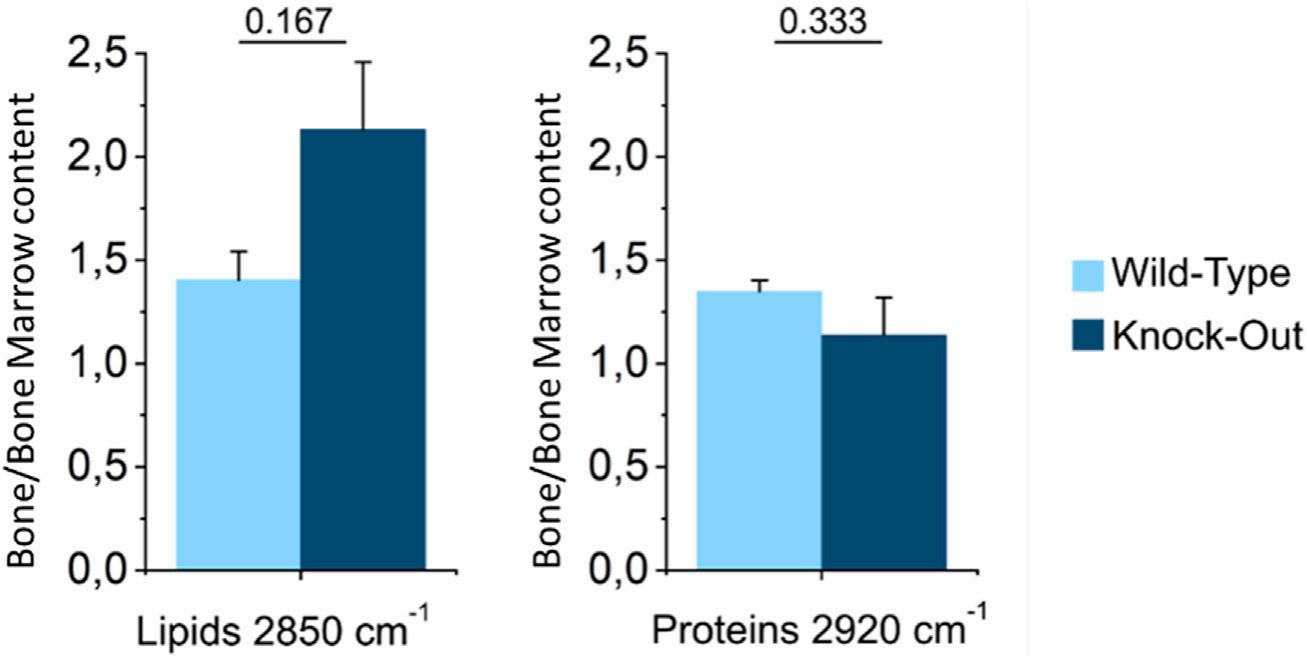
Ratio between the average signal intensity in the bone and in the bone marrow for lipids and proteins in WT and KO models. Statistical analysis was performed using *t* test. *p*-values are indicated above each bar plot (*n* = 3 for both genotypes).

**FIGURE 8:**
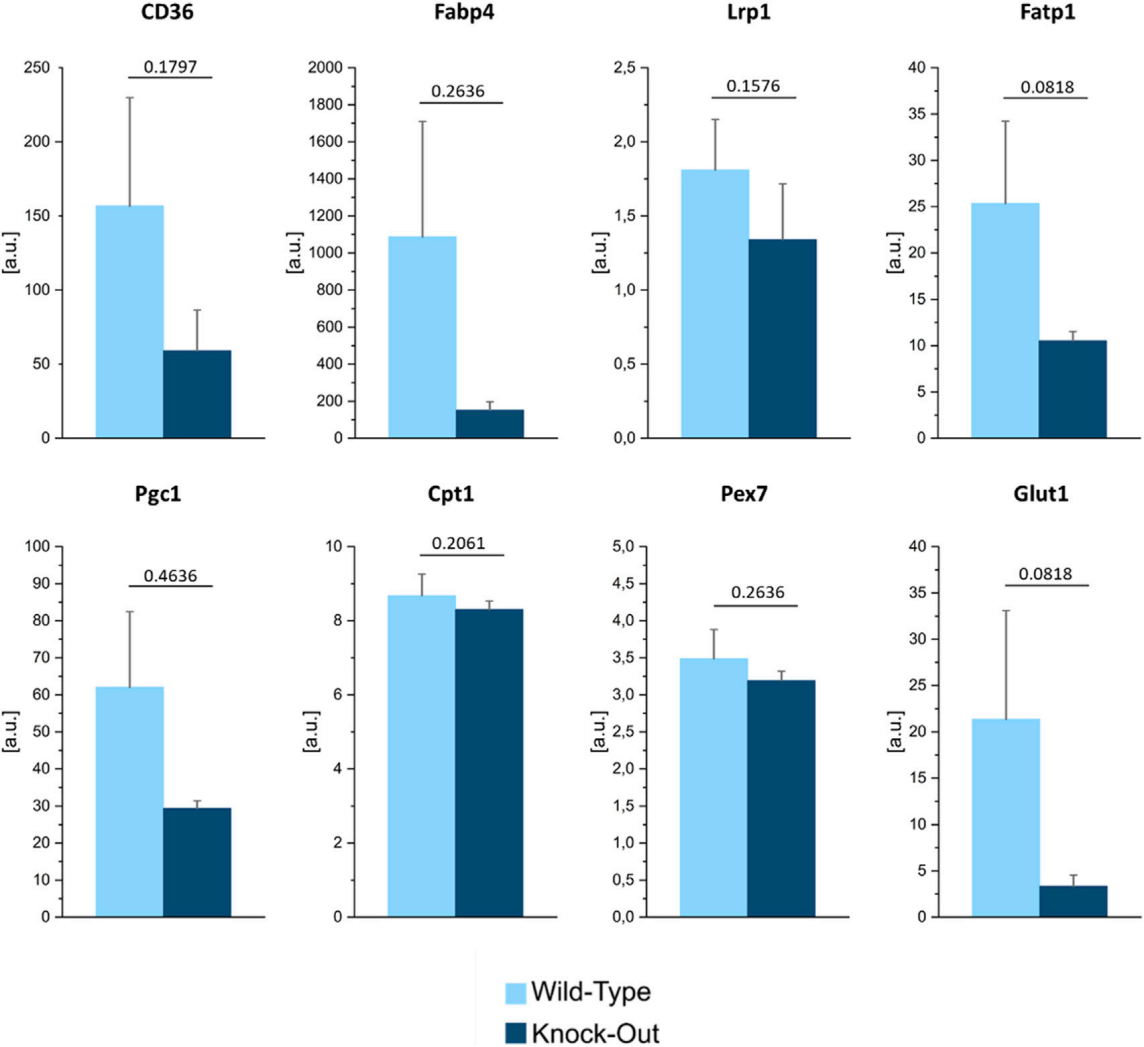
Gene expression analysis of selected genes relevant for lipid transport, uptake, and metabolism, in the flushed bone of WT and Dpp3 KO mice (*n* = 4 WT and 7 KO). Statistical analysis was performed using Mann Whitney test; *p* values are indicated above each graph.

To quantify these differences, we plotted the average lipid and protein signals registered in the bone normalized over the ones collected in the bone marrow, for both the WT and KO samples (Figure 7). The ratios were larger than one for both genotypes, indicating that, in general, bone contains more proteins and lipids compared to the bone marrow. For what pertains to the lipid signal, the bone/bone-marrow ratio was greater in KO *vs.* WT samples. This observation may point to a different skeletal metabolism in the presence or absence of Dpp3. Of note, increasing evidence in literature shows the importance of cellular metabolism in the molecular control of skeletal cell functions, and the association between metabolic dysregulation and skeletal degenerative diseases and ageing (Van Gastel and Carmeliet, 2021). Therefore, to support the hypothesis raised by SRS analysis of lipids, we assessed the expression of genes related to lipid transport (CD36, Fabp4, and Fatp1), uptake (Fabp4, Lrp1) and utilization (Cpt1), and in energy metabolism (Pgc1, Pex7, and Glut1) in the flushed bone of WT and Dpp3 KO mice by qPCR and found an altered expression pattern in the KO (Figure 8). Overall, these data add to the hypothesis of altered bone metabolism in the absence of DPP3 raised by SRS analysis. In conclusion, a non-conventional analysis of bone uncovered a possible metabolic alteration in this tissue in the absence of Dpp3. A recent clinical study proposed DPP3 as a bone protective factor, by showing significant association with femoral neck bone mineral density in post-menopausal osteoporotic women before treatment and significant reduction in patients compared to controls (Menale et al., 2022). Taken together, these findings will deserve further investigation, also considering their possible translational relevance for human bone pathophysiology.

## 4 Conclusion

We demonstrated multimodal NLO microscopy as a quantitative, chemically selective, and non-destructive tool to effectively reveal features of bone biochemical composition and morphological arrangement in label-free murine spines, imaging large tissue areas that include complete vertebrae. This advanced optical technology allowed highlighting changes in terms of collagen amount and orientation, along with modifications in the lipid content between tissue samples from a WT and a mutant mouse (Dpp3 KO), the latter modeling a bone loss condition relevant to human pathology.

Our work provides a proof of concept of the application of multimodal NLO microscopy on entire spine tissue sections to derive typical traits of pathophysiological bone conditions. The quantitative nature of this type of microscopy data is also well suited for computational methods of automatic detection and classification, in the framework of artificial intelligence-driven diagnostics. Also, the presented multimodal NLO microscope was adapted to scan large tissue areas, thus collecting a considerable amount of data per acquired multichannel image, which would serve the need for a rich training dataset to boost computational accuracy. By coupling label-free multimodal NLO imaging, not requiring any time-consuming sample preparation, to machine learning models, trained to predict in real-time the probability of bone loss diseases, one can offer a rapid and accurate system to aid and augment clinical decisions in bone histopathology protocols. Validation of this analytical approach on diverse pathological samples will corroborate our conclusion and pave the way to improvement of the standard histopathological and immunohistochemical practice in bone biomedical analysis, through the prospective introduction of advanced NLO microscopy tools in clinical diagnostics (Scott, 1979; Sobel and Feldman, 2015; Gaytan et al., 2020).

## Data Availability

The raw data supporting the conclusion of this article will be made available by the authors, without undue reservation.
