# Supplementary material for: Label-free multimodal nonlinear optical microscopy reveals features of bone composition in pathophysiological conditions
Source: Front Bioeng Biotechnol. 2022 Nov 22;10:1042680. doi: 10.3389/fbioe.2022.1042680 (PMC9723390; doi:10.3389/fbioe.2022.1042680)
Supplement: Supplementary file 1 [file Table1.DOCX]

**SUPPLEMENTARY INFORMATION**

**Label-free multimodal nonlinear optical microscopy reveals hallmarks of bone composition in pathophysiological conditions**

Benedetta Talone†^1^, Arianna Bresci†^1^, Francesco Manetti^1^, Federico Vernuccio^1^, Alejandro De la Cadena^1^, Chiara Ceconello^1^, Maria Lucia Schiavone^2^, Stefano Mantero^2,3^, Ciro Menale^4^, Renzo Vanna^5^, Giulio Cerullo^1,5^, Cristina Sobacchi^2,3*^ and Dario Polli^1,5*^

^1^Department of Physics, Politecnico di Milano, P.zza Leonardo da Vinci 32, 20133 Milan, Italy

^2^IRCCS Humanitas Research Hospital, via Manzoni 56, 20089 Rozzano (Mi), Italy

^3^ CNR-Institute for Genetic and Biomedical Research (CNR-IRGB), via Fantoli 16/15, 20138 Milan, Italy

^4^Department of Clinical Medicine and Surgery, University of Naples "Federico II", via Pansini 5, 80131 Naples, Italy

^5^CNR-Institute for Photonics and Nanotechnologies (IFN-CNR), P.zza Leonardo Da Vinci 32, 20133 Milan, Italy

†These authors contributed equally to this work and share first authorship

* Corresponding Authors: [cristina.sobacchi@humanitasresearch.it](mailto:cristina.sobacchi@humanitasresearch.it) and [dario.polli@polimi.it](mailto:email@uni.edu)

**Supplementary Figure 1**


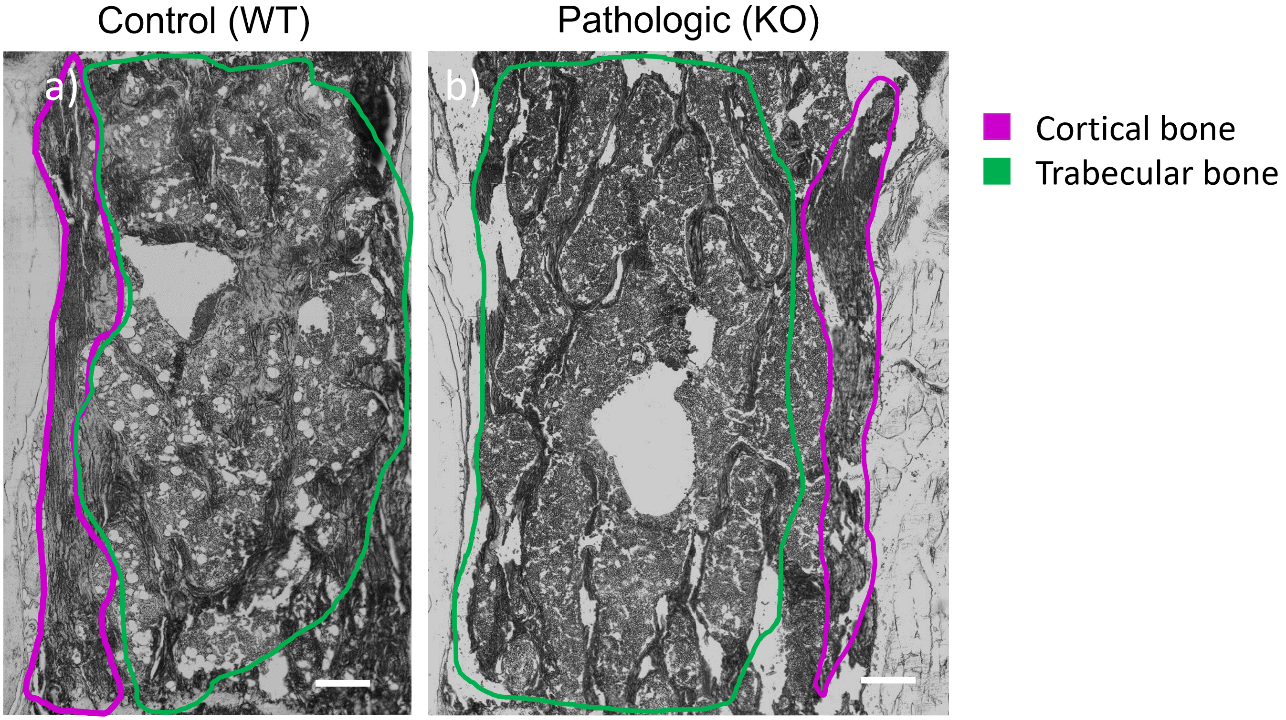


Transmission images of control (a) and pathologic (b) murine spine sections. The cortical region and the trabecular region, filled with bone marrow, are evidenced in purple and in green, respectively. The scale bar is 200 µm.

**Supplementary Figure 2**

**
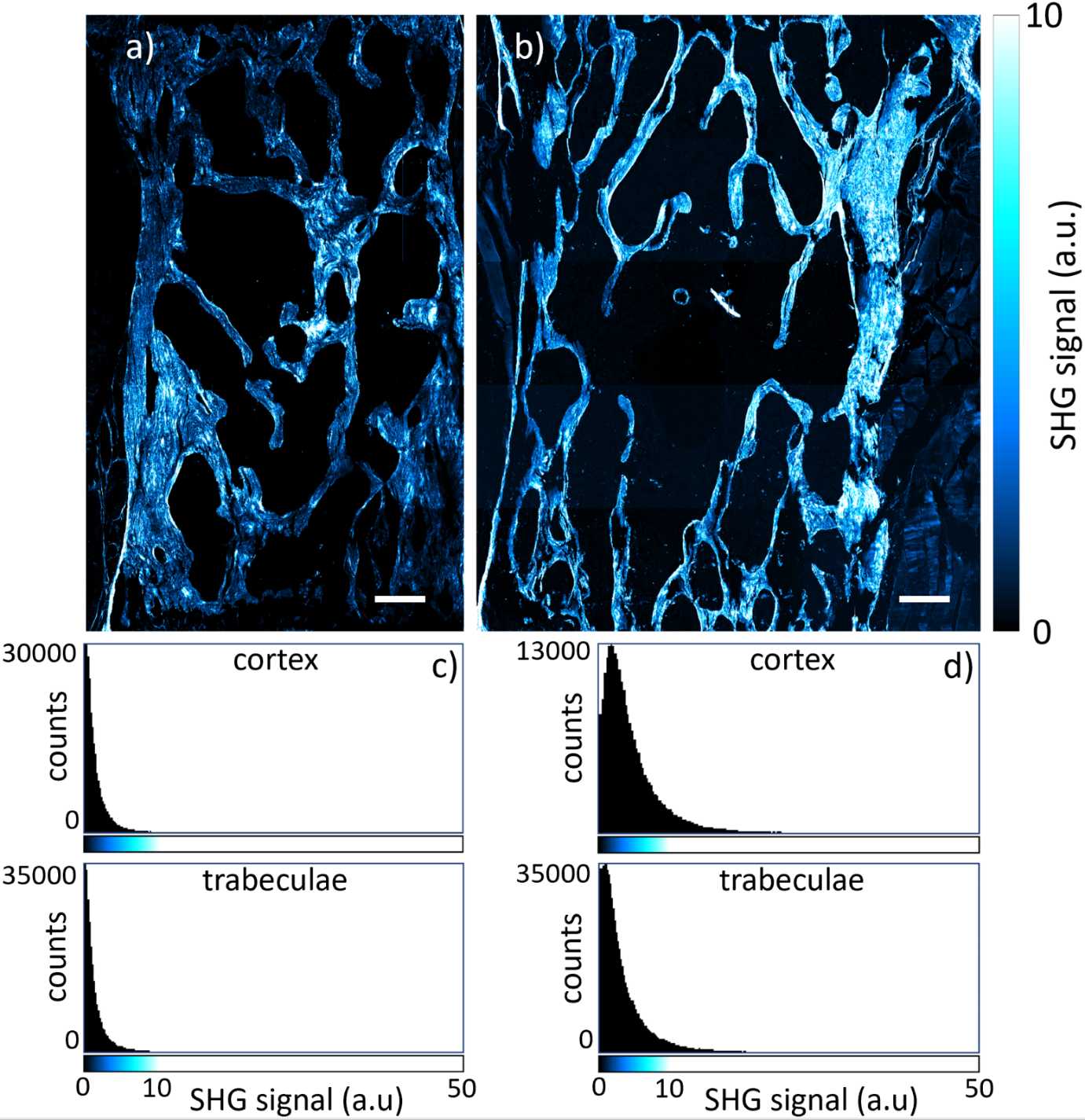
**

SHG imaging of control (a) and pathologic (b) murine spine sections, via a parallel-polarized excitation field, showcases differences in terms of signal intensity between the two samples. As SHG is linearly dependent on the scatterers concentration, this, in turn, implies changes in the collagen fibrils density, coherently with the osteoporosis condition induced in the KO model. Coherently with the results presented in Fig. 4, the bins of the pathologic data (d) are shifted to the right, *i.e.* to higher values, compared to the control case (c), both in the cortex and in the trabeculae ROIs, with a significant portion of pixels yielding signals well above 10 a.u. Moreover, the bins in (d) are distributed on a wider range of values: taking into consideration the parallel-polarized excitation field, this evidence seemingly suggests that the collagen fibers are more randomly oriented in the *Dpp3* KO mice section. The scale bar is 200 µm.
